# Association between multimorbidity and mortality in a cohort of patients admitted to hospital with COVID-19 in Scotland

**DOI:** 10.1177/01410768211051715

**Published:** 2021-10-21

**Authors:** Utkarsh Agrawal, Amaya Azcoaga-Lorenzo, Adeniyi Francis Fagbamigbe, Eleftheria Vasileiou, Paul Henery, Colin R Simpson, Sarah J Stock, Syed Ahmar Shah, Chris Robertson, Mark Woolhouse, Lewis D Ritchie, Aziz Shiekh, Ewen M Harrison, Annemarie B Docherty, Colin McCowan

**Affiliations:** 1 School of Medicine, University of St. Andrews, KY16 9TF, UK; 2 Usher Institute, The University of Edinburgh, Edinburgh, EH8 9YL, UK; 3 MRC/CSO Social and Public Health Sciences Unit, University of Glasgow, Glasgow, G3 7HR, UK; 4 Victoria University of Wellington, School of Health, Wellington Faculty of Health, Wellington PO Box 600,Wellington 6140, New Zealand; 5 Department of Mathematics and Statistics, University of Strathclyde, Glasgow, G1 1XQ, UK; 6 Academic Primary Care, University of Aberdeen, Aberdeen, AB24 3FX, UK; 7 Department of Clinical Surgery, The University of Edinburgh, Edinburgh, EH16 4SA, UK

**Keywords:** COVID-19, SARS-CoV-2, hospital admissions, multimorbidity, shielding

## Abstract

**Objectives:**

We investigated the association between multimorbidity among patients hospitalised with COVID-19 and their subsequent risk of mortality. We also explored the interaction between the presence of multimorbidity and the requirement for an individual to shield due to the presence of specific conditions and its association with mortality.

**Design:**

We created a cohort of patients hospitalised in Scotland due to COVID-19 during the first wave (between 28 February 2020 and 22 September 2020) of the pandemic. We identified the level of multimorbidity for the patient on admission and used logistic regression to analyse the association between multimorbidity and risk of mortality among patients hospitalised with COVID-19.

**Setting:**

Scotland, UK.

**Participants:**

Patients hospitalised due to COVID-19.

**Main outcome measures:**

Mortality as recorded on National Records of Scotland death certificate and being coded for COVID-19 on the death certificate or death within 28 days of a positive COVID-19 test.

**Results:**

Almost 58% of patients admitted to the hospital due to COVID-19 had multimorbidity. Adjusting for confounding factors of age, sex, social class and presence in the shielding group, multimorbidity was significantly associated with mortality (adjusted odds ratio 1.48, 95%CI 1.26–1.75). The presence of multimorbidity and presence in the shielding patients list were independently associated with mortality but there was no multiplicative effect of having both (adjusted odds ratio 0.91, 95%CI 0.64–1.29).

**Conclusions:**

Multimorbidity is an independent risk factor of mortality among individuals who were hospitalised due to COVID-19. Individuals with multimorbidity could be prioritised when making preventive policies, for example, by expanding shielding advice to this group and prioritising them for vaccination.

## Introduction

As of 9 June 2021, severe illness among the patients infected with severe acute respiratory syndrome coronavirus 2 (SARS-CoV-2) has led to more than 3.5 million deaths^1^ across the globe, including almost 128,000 deaths in the UK. Although at this point in time, vaccination programmes have successfully been rolled out in a majority of rich countries leading to a significant reduction in the number of cases and fatalities,^2,3^ most of the population around the world remains unvaccinated and still at risk.^4^ Until vaccines are not widely distributed, it is crucial that we continue researching and reporting on what are the factors associated with worse outcomes to allow mitigation strategies and adequate protection of those deemed vulnerable to be provided.

It is known that almost 15% of individuals infected with SARS-CoV-2 need hospitalisation and mortality is reported among 30% of hospitalised patients.^5,6^ There is evidence from previous work showing that the risk of severe outcomes increases with different factors, including increasing age age,^7^ male sex^8^ and the presence of specific pre-existing long-term conditions (such as hypertension, diabetes or chronic kidney disease, etc.).^9–11^ Multimorbidity (defined as two or more long-term conditions) is strongly associated with age and is very prevalent in both high- and low-middle income countries.^12^ The importance and impact of multimorbidity on subsequent mortality among patients admitted to hospital with COVID-19 is under researched; however, two previous publications have shown that the Charlson index of comorbidity is a good predictor of mortality in cases of severe COVID-19-related infections.^13,14^

In the UK, following the evidence from the initial reports showing a higher risk of adverse outcomes associated with certain conditions, a Shielded Patient List was created.^10^ This list included a record of vulnerable patients with specific conditions or treatments thought to be at high risk of complications from COVID-19.^15^ Individuals having any condition on the Shielded Patient List received advice to avoid or minimise non-essential contacts. However, no special recommendation was made to patients with multimorbidity if they did not have any of those conditions. Understanding how multimorbidity impacts on patients admitted to a hospital due to severe COVID-19 is important to help clinicians with the management of these patients and provide meaningful analysis for public health policymakers around the world.^9,16–18^

We aimed to investigate how multimorbidity, Shielded Patient List conditions and demographic factors including age, sex and socio-economic deprivation in patients hospitalised with COVID-19 was associated with an increased mortality risk during the first wave (28 February to 22 September 2020) of the pandemic in Scotland. Specific research questions were: (1) What was the prevalence of multimorbidity and how it varied by age, sex and deprivation among the population admitted to the hospital with COVID-19?, (2) What was the association between COVID-19-related mortality and multimorbidity, and how it varied by age, sex and deprivation? and (3) Was there an increased risk of mortality from COVID-19 if patients were on the Shielded Patient List and multimorbid?

## Materials and methods

### Study design and data sources

Patients admitted to Scottish hospitals with COVID-19 were identified through the electronic communication of surveillance in scotland (ECOSS) and hospital admissions (SMR01) database. We included patients with an admission within 14 days of PCR + ve test or validated by the presence of ICD-10 codes U07.1 and U07.2. Using routinely collected secondary care data, we developed a cohort of 4684 patients hospitalised in Scotland due to COVID-19 for the first time between 28 February 2020 and 22 September 2020. We retrieved Scottish national data on outpatient appointment and attendances (SMR00), hospital admissions (SMR01), Community Prescriptions (PIS), Electronic Communication of Surveillance in Scotland and Death Registrations and Demography.^19^ The datasets used the Community Health Index (CHI) numbers that are present on all healthcare encounters in Scotland to link the datasets, which were then anonymised and made available on the National Data Safe Haven platform.^19,20^

### Variables

#### Exposure

The primary independent variable was the presence of multimorbidity. To identify the conditions of each patient for multimorbidity analysis, we used data from routinely collected health records from 1 April 2006. To define the presence of multimorbidity, we used all 31 conditions included in the Elixhauser comorbidity Index^21,22^ and the eight conditions from nine listed in the Shielded Patient List^15^ (further details on all the conditions can be found in Supplementary Material). We were unable to identify pregnant women from the available data who were advised to shield. Levels of multimorbidity were categorised as ‘no multimorbidity’ (no recorded condition or only one condition) and ‘multimorbidity’ (for 2 + recorded conditions). Additionally, we also carried out an analysis of ‘complex multimorbidity’, defined as the presence of four or more recorded conditions.

#### Outcome

We identified mortality among patients admitted to hospital for COVID-19 as reported via National Records of Scotland death certificates coded for COVID-19 on the death certificate (U07.1 and U07.2 ICD-10 codes) or death within 28 days of a positive COVID-19 test.

### Other variables

Other explanatory variables included in the model based on their known relationship to multimorbidity and death were patient’s sex (male or female), age group (<50, 51–65, 66–80 and >80 years) and the level of deprivation measured by quintiles (most deprived to least deprived) based on the Scottish Index of Multiple Deprivation attributed to the home postcode of the patient. We merged under 18 s with the age group 19–50 to safeguard against potential unintended disclosure risk for individuals due to small numbers.

### Statistical analysis

Descriptive statistics reported the prevalence of multimorbidity among individuals hospitalised due to COVID-19 and its variation by different demographic characteristics. Numbers and proportions within each group of interest were reported.

A logistic regression model, with clustering of patients within the hospital, was used to assess the risk of death among COVID-19 hospitalised patients and the presence of multimorbidity among these patients. An univariable regression model was used to analyse the risk of mortality against the presence of multimorbidity, presence in the Shielded Patient List and demographic characteristics including age, sex and deprivation. A multivariable regression model adjusting for all the significant aforementioned factors was used. Adjusted odds ratio (AOR), and 95% confidence interval (CI) were reported. Although outcomes were expected to be relatively common, reporting OR was still a valid approach.^23^ We also analysed a random-effects model to check for clustering of patients within the hospital.

Since previous studies associated mortality with increasing comorbidities and presence in the Shielded Patient List,^24^ we explored the interaction between them and the prediction on mortality. Subsequently, a multivariable logistic regression model tested for interaction between the presence of multimorbidity and the presence in the Shielded Patient List, in the prediction of mortality.

### Ethics, software and dissemination

We obtained approval from Scotland A Research Ethics Committee (Ref: 20/SS/0028) and Public Benefit and Privacy Panel for Health and Social Care (HSC-PBPP). R Studio statistical software (R version 3.60)^25^ was used for all the analysis and visualisation. All the R codes will be published on Github.

### Reporting guidelines

Guidelines from Reporting of studies Conducted using Observational Routinely-collected Data (RECORD) extended from the Strengthening the Reporting of Observational Studies in Epidemiology (STROBE) was used to communicate the findings.

## Results

Of the 6512 individuals hospitalised between 28 February 2020 and 22 September 2020 in Scotland due to COVID-19, we could link and validate information for 4684 (71.9%) individuals and thus included all these individuals in the study. The median age of patients was 71 years (interquartile range 57–82). Most patients admitted to hospital with COVID-19 were affected by multimorbidity (2713, 57.9%) and almost half of this group had complex multimorbidity (1248, 26.6%). There were 1330 (28.4%) recorded deaths. Among individuals who died, more than three quarters (989, 74.4%) had multimorbidity and almost four in 10 had complex multimorbidity (503, 40.3%).

### Description of COVID-19-related hospitalisation and prevalence of multimorbidity

**Figure 1.**
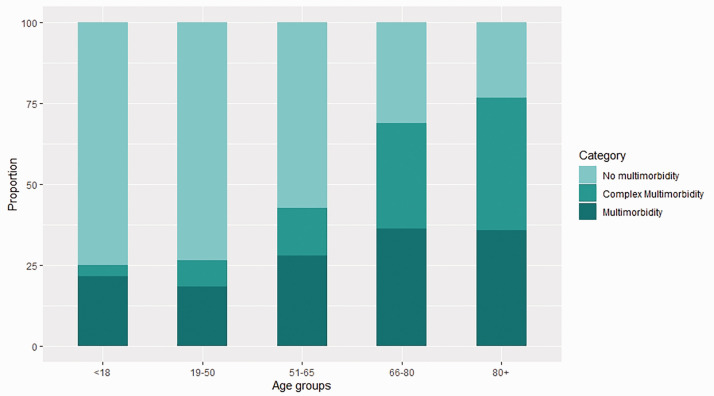
Distribution of individuals with no multimorbidity, multimorbidity and complex multimorbidity within each age groups. The proportion of no multimorbidity in each age band decreased with age while that of complex multimorbidity increased. For visualisation, no multimorbidity was ≤1 conditions, multimorbidity was described as 2–3 conditions, and complex multimorbidity was 4+.

The proportion of patients with multimorbidity increased with age from 26.5% in the under 50 age group to 76.7% among patients aged 80 years or higher (see Figure 1), but there was little difference by sex (Table 1). There were higher levels of multimorbidity and complex multimorbidity (81.6%, 45.0%) for people in the Shielded Patient List than in the non-shielding group (43.7%, 15.6%). Lastly, there was also social patterning of multimorbidity where it was more prevalent in people from deprived areas.

**Table 1.** Distribution of multimorbidity by patients characteristics for hospital admissions due to COVID-19.

		Number of conditions (*n* (%))		
Characteristics	n	No multimorbidity (≤1)	Multimorbidity (2+)	Complexmultimorbidity (4+)
Hospitalisation	4684	1971 (42.1)	2713 (57.9)	1248 (26.6)
Age group (years)				
<50	688	506 (73.5)	182 (26.5)	55 (8.0)
51–65	1234	709 (57.5)	525 (42.5)	182 (14.7)
66–80	1457	452 (31.0)	1005 (69.0)	476 (32.7)
80+	1305	304 (23.3)	1001 (76.7)	535 (41.0)
Sex				
Male	2531	1087 (42.9)	1444 (57.1)	647 (25.6)
Female	2151	883 (41.1)	1268 (58.9)	601 (27.9)
Missing	2			
Shielding group				
Yes	1759	324 (18.4)	1435 (81.6)	792 (45.0)
No	2925	1647 (56.3)	1278 (43.7)	456 (15.6)
Deprivation				
Most	1320	483 (36.6)	837 (63.4)	409 (31.0)
2	1058	436 (41.2)	622 (58.8)	299 (28.3)
3	833	339 (40.7)	494 (59.3)	214 (25.7)
4	778	357 (45.9)	421 (54.1)	177 (22.8)
Least	679	349 (51.4)	330 (48.6)	147 (21.6)
Missing	16	–	–	–

### Mortality related to multimorbidity and other patient characteristics

**Figure 2.**
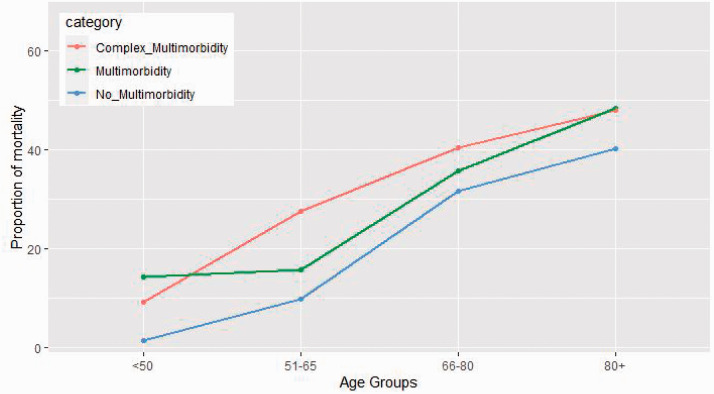
Variation of mortality proportion by age groups for individuals with no multimorbidity, multimorbidity and complex multimorbidity. For visualisation, no multimorbidity was ≤1 conditions, multimorbidity was described as 2–3 conditions, and complex multimorbidity was 4+.

COVID-19-related mortality was high among patients with multimorbidity (989, 36.5%, see Table 2). Over half of these patients had complex multimorbidity with a higher proportion of mortality in this group (503, 40.3%). Mortality increased with age from 4.4% for patients under the age of 50 to 46.1% in patients aged 80 years or older (see Figure 2). Almost half of the hospitalised patients were males, and they had a higher mortality (32.2%) in comparison to females (24.0%). Among patients in the shielding group and non-shielding group, the proportion of mortality was 36.8% and 23.4%, respectively.

**Table 2.** Distribution of hospitalisation and mortality by patient characteristics including multimorbidity.

	All (*n* (%))	Number of deaths	Mortality (%)
	4684	1330	28.4
Multimorbidity			
No (≤1)	1971 (42.1)	341	17.3
Yes (2+)	2713 (57.9)	989	36.5
4+	1248 (26.6)	503	40.3
Age group			
<50	688 (14.7)	30	4.4
51–65	1234 (26.3)	173	14.0
66–80	1457 (31.1)	526	36.1
80+	1305 (27.9)	601	46.1
Sex			
Male	2531 (54.0)	814	32.2
Female	2151 (45.9)	516	24.0
Missing	2 (0.1)	–	
Shielding group			
Yes	1759 (37.6)	647	36.8
No	2925 (62.4)	683	23.4
Deprivation			
Most	1320 (28.2)	389	29.5
2	1058 (22.6)	291	27.5
3	833 (17.8)	263	31.6
4	778 (16.6)	214	27.5
Least	679 (14.6)	171	25.2
Missing	16 (0.3)	<5	–

### Association of mortality with multimorbidity

Patients with multimorbidity, increasing age, males and present in Shielded Patient List were associated with increased risk of death. Controlling for age, sex and deprivation, it was observed that patients with multimorbidity were at higher risk of death than those without it (AOR 1.48, 95%CI 1.26–1.75, *p* < 0.001) (see Table 3). Mortality increased with age significantly and was almost 15 times (AOR15.34, 95% CI 10.50–23.26, *p* < 0.001) higher among those aged 80 years or more than those aged 19 to 50 years.

**Table 3.** Odds ratio of univariable and multivariable models for risk of death adjusted for patient characteristics including multimorbidity.

Factors	Unadjsuted odds ratio (95% CI, *p*-value)	Adjusted odds ratio (95% CI, *p*-value)
Multimorbidity		
No	1.00	1.00
Yes	2.70 (2.34–3.11, *p* < 0.001)	1.49 (1.26–1.75, *p* < 0.001)
Age group		
19–50	1.00	1.00
51–65	3.41 (2.29–5.09, *p* < 0.001)	3.14 (2.09–4.72, *p* < 0.001)
66–80	11.82 (8.07–17.33 *p* < 0.001)	9.41 (6.35–13.96, *p* < 0.001)
80+	17.93 (12.23–26.30, *p* < 0.001)	15.34 (10.31–22.80, *p* < 0.001)
Sex		
Female	1.00	1.00
Male	1.50 (1.32–1.71, *p* < 0.001)	1.72 (1.49–1.98, *p* < 0.001)
Shielding		
No	1.00	1.00
Yes	1.89 (1.66 – 2.15, *p* < 0.001)	1.31 (1.13–1.52, *p* < 0.001)
Deprivation		
Most	1.24 (1.00–1.54, *p* = 0.055)	1.36 (1.08–1.71, *p* = 0.010)
2	1.13 (0.91–1.42, *p* = 0.270)	1.19 (0.94–1.51, *p* = 0.152)
3	1.35 (1.07–1.70, *p* = 0.011)	1.42 (1.11–1.82, *p* = 0.005)
4	1.11 (0.88–1.41, *p* = 0.389)	1.18 (0.91–1.52, *p* = 0.205)
Least	1.00	1.00

Since no death was reported in the under 18 age groups, it was excluded from the modelling analysis and to safeguard against potential unintended disclosure risk for individuals. We also grouped patients by the hospital in which they were admitted to check for any random effect, and there were very small random effects (variance: 0.008, CI: 0.006–0.010).

### Interaction analysis

Since shielding had a strong positive association with multimorbidity, we explored interaction effects between multimorbidity and being in the shielding group but found no association with a multiplicative effect on risk of mortality among the cohort (see Supplementary Material Table 1).

## Discussion

Our analysis showed that in patients admitted to hospital due to COVID-19, 6 out of 10 had multimorbidity, with almost half of this group having complex multimorbidity. The presence of multimorbidity was associated with a 50% increase in the risk of death, after adjusting for age, sex, social class and shielding status. Patients who had been advised to shield were at higher risk of mortality from COVID-19 than those not shielding and this was independent of the effect of multimorbidity.^26–28^

Strengths of the work included the prospective data collection in a standardised manner across a number of hospitals. However, one limitation of the study is that data were not available on the ethnicity of all the patients, so it was not included as a confounder. The use of the Elixhauser comorbidity index based on electronic health records^21,22^ combined with information on conditions from the shielding list^15^ provided a robust and transparent mechanism to determine multimorbidity. Although only selecting the aforementioned comorbidities based on previous secondary care admissions is a limitation of the study, they incorporate a core set of disorders for multimorbidity measurement that covers a wide range of conditions including a total of 39 different conditions. Moreover, using the secondary care data, we were able to capture the most severe patients within the multimorbidity spectrum. The level of multimorbidity will change dependent on which list of conditions is included and the consequences of multimorbidity also change with different combinations and severity of conditions, which we did not explore in this analysis. Another limitation of the study is that primary care data were not available, which may have resulted in underestimates of the prevalence of multimorbidity as it was based on secondary care data.

Our findings reinforce that the presence of multimorbidity is associated with the risk of death among individuals hospitalised with COVID-19. Some previous work have shown the association of few individual comorbidities (which are generally Shielded Patient List conditions) with adverse outcomes,^16,13,29^ we included an expanded list of 39 conditions. Although deprivation was not found to be a significant factor for patients with worse outcomes in this analysis, among the patients admitted to the hospital, the proportion increased with the deprivation index. Some previous studies have reported that individuals in the Shielded Patient List were at a very high risk of severe outcomes.^24,30^ In addition, we also found that these conditions were a risk factor independent of the presence of multimorbidity.

This study suggests that multimorbidity should be considered as an independent risk factor of worse outcomes in the case of contracting COVID-19. This population should thus be prioritised when protective and preventive measures are planned like expanding shielding advice or vaccination programs. Social support should also be provided to this population for them being able to protect themselves. Moreover, the results also indicate that the advice given to shielding patients based on individual conditions could also be extended to those with multimorbidity and especially complex multimorbidity as they have similarly high levels of mortality. The effect of age on mortality for patients with COVID-19 is well established, but our analysis of multimorbidity by age suggests younger people with high levels of multimorbidity may be at similar levels of risk as older patients.

## Conclusion

Multimorbidity is a risk factor for mortality in patients admitted to hospital with COVID-19 and the risk increases with higher levels of multimorbidity. Patients who were advised to shield to protect themselves from COVID-19 were also at higher risk of mortality, and this is independent of multimorbidity. We suggest that the advice given to shielding patients could be extended to those with multimorbidity and especially complex multimorbidity as they have similarly high levels of mortality.

## Supplementary Material

Supplementary material

## Declarations

**Competing Interests:** The author(s) declared the following potential conflicts of interest with respect to the research, authorship, and/or publication of this article: AS is a member of the Editorial Board of the Journal of Royal Society of Medicine, the Scottish Government Chief Medical Officer’s COVID-19 Advisory Group, the Scottish Government Standing Committee on Pandemics, and the New and Emerging Respiratory Virus Threats (NERVTAG) Risk Stratification Subgroup. MW is also a member of C19AG, and also the SAGE subcommittee SPI-M. CRS declares funding from the MRC, NIHR, CSO and New Zealand Ministry for Business, Innovation and Employment and Health Research Council during the conduct of this study. All other authors have no other competing interests to declare.

**Ethics approval:** Not applicable.

**Guarantor:** UA.

**Contributorship:** CMC, ABD, EMH, HS and UA conceived this study. UA led the analysis with the help of AFF. CMC, EMH, ABD and AAL advised on the analysis plans. All authors contributed to the study design. UA led the draft writing with the help of AAL, AFF and CMC. All the authors contributed to drafting the protocol and the revised manuscript for important intellectual content. All authors gave final approval of the version to be published.

**Data availability:** All the codes and figures will be made available on EAVE-II Github (https://github.com/EAVE-II/multimorbidity-and-mortality-hospital-admission)
