# Supplementary material for: Association between multimorbidity and mortality in a cohort of patients admitted to hospital with COVID-19 in Scotland
Source: J R Soc Med. 2021 Oct 21;115(1):22–30. doi: 10.1177/01410768211051715 (PMC8811325; doi:10.1177/01410768211051715)
Supplement: Supplementary material [file sj-pdf-1-jrs-10.1177_01410768211051715.pdf]

Association between multimorbidity and mortality in a cohort of patients admitted to hospital with COVID-19 in Scotland

Supplementary material

Power Calculation: A power calculation was performed to check if we were adequately powered to rely on the interaction estimates. At alpha 0.05 and power 0.8, we only needed 500 samples and we have 5401 samples so we are adequately powered for the interaction analysis.

List of 39 conditions included in the analysis

Conditions in the Shielded Patient List (SPL)[1]

1. solid organ transplant
2. Severe respiratory conditions including all cystic fibrosis, severe asthma and severe chronic obstructive pulmonary (COPD)
3. Rare diseases and inborn errors of metabolism that significantly increase the risk of infections (such as Severe combined immunodeficiency (SCID), homozygous sickle cell)
4. Immunosuppression therapies sufficient to significantly increase risk of infection
5. Spleen
6. Down's syndrome
7. Stage 5 Chronic Kidney Disease
8. people with cancer who are undergoing active chemotherapy

List of Conditions in the Elixhauser Comorbidity Index[2]

1. Congestive heart failure
2. Cardiac arrhythmias
3. Valvular disease
4. Pulmonary circulation disorders
5. Peripheral vascular disorders
6. Hypertension, uncomplicated
7. Hypertension, complicated
8. Paralysis
9. Other neurological disorders
10. Chronic pulmonary disease
11. Diabetes, uncomplicated
12. Diabetes, complicated
13. Hypothyroidism
14. Renal failure
15. Liver disease
16. Peptic ulcer disease, excluding bleeding

[1] women who are pregnant were not included due to lack of GP data or SMR02
[2] All the codes that were part of the SPL list were removed from the Elixhauser Comorbidity Index List

17. AIDS/HIV
18. Lymphoma
19. Metastatic cancer
20. Solid tumour without metastasis
21. Rheumatoid arthritis/collagen vascular diseases
22. Coagulopathy
23. Obesity
24. Weight loss
25. Fluid and electrolyte disorders
26. Blood loss anaemia
27. Deficiency anaemia
28. Alcohol abuse
29. Drug abuse
30. Psychoses
31. Depression

Table 1: Multivariable models analysing the interaction between presence in shielding group and multimorbidity against mortality

**Factors**	**AOR without interaction (95% CI)**	**AOR for interaction (95% CI)**
**Age Group**		
19-50	1.00	1.00
51-65	3.14 (2.09-4.72, p<0.001)	3.14 (2.08-4.72, p<0.001)
66-80	9.41 (6.35-13.96, p<0.001)	9.40 (6.34-13.94, p<0.001)
80+	15.34 (10.31-22.80, p<0.001)	15.31 (10.29-22.76, p<0.001)
**Sex**		
Female	1.00	1.00
Male	1.72 (1.49-1.98, p<0.001)	1.72 (1.49-1.98, p<0.001)
**Deprivation**		
Most	1.36 (1.08-1.71, p=0.010)	1.36 (1.08-1.71, p=0.010)
2	1.19 (0.94-1.51, p=0.152)	1.19 (0.94-1.51, p=0.152)
3	1.42 (1.11-1.82, p=0.005)	1.42 (1.11-1.82, p=0.005)
4	1.18 (0.91-1.52, p=0.205)	1.18 (0.91-1.52, p=0.204)
Least	1.00	1.00
**Multimorbidity**		
No	1.00	1.00
Yes	1.49 (1.26-1.75, p<0.001)	1.53 (1.27-1.88, p<0.001)
**Shielding**		
No	1.00	1.00
Yes	1.31 (1.13-1.52, p<0.001)	1.43 (1.05-1.95, p=0.023)
**Interaction**	-	1.00
Multimorbidity-shielding		0.89 (0.63 – 1.27, p=0.533)
